# Paraventricular oxytocin neurons impact energy intake and expenditure: projections to the bed nucleus of the stria terminalis reduce sucrose consumption

**DOI:** 10.3389/fendo.2024.1449326

**Published:** 2024-09-02

**Authors:** Amy A. Worth, Claire H. Feetham, Nicole A. Morrissey, Simon M. Luckman

**Affiliations:** Faculty of Biology, Medicine and Health, University of Manchester, Manchester, United Kingdom

**Keywords:** oxytocin, paraventricular nucleus, chemogenetic, optogenetic, BNST, sucrose, food intake

## Abstract

**Background:**

The part played by oxytocin and oxytocin neurons in the regulation of food intake is controversial. There is much pharmacological data to support a role for oxytocin notably in regulating sugar consumption, however, several recent experiments have questioned the importance of oxytocin neurons themselves.

**Methods:**

Here we use a combination of histological and chemogenetic techniques to investigate the selective activation or inhibition of oxytocin neurons in the hypothalamic paraventricular nucleus (Oxt^PVH^). We then identify a pathway from Oxt^PVH^ neurons to the bed nucleus of the stria terminalis using the cell-selective expression of channel rhodopsin.

**Results:**

Oxt^PVH^ neurons increase their expression of cFos after both physiological (fast-induced re-feeding or oral lipid) and pharmacological (systemic administration of cholecystokinin or lithium chloride) anorectic signals. Chemogenetic activation of Oxt^PVH^ neurons is sufficient to decrease free-feeding in *Oxt* ^Cre:hM3Dq^ mice, while inhibition in *Oxt* ^Cre:hM4Di^ mice attenuates the response to administration of cholecystokinin. Activation of Oxt^PVH^ neurons also increases energy expenditure and core-body temperature, without a significant effect on locomotor activity. Finally, the selective, optogenetic stimulation of a pathway from Oxt^PVH^ neurons to the bed nucleus of the stria terminalis reduces the consumption of sucrose.

**Conclusion:**

Our results support a role for oxytocin neurons in the regulation of whole-body metabolism, including a modulatory action on food intake and energy expenditure. Furthermore, we demonstrate that the pathway from Oxt^PVH^ neurons to the bed nucleus of the stria terminalis can regulate sugar consumption.

## Introduction

Much has been written about the neurohormone oxytocin and metabolism, though the actual role of oxytocin and oxytocin receptors in the normal, physiological regulation of feeding is keenly debated (see recent reviews (1–3)). There is little doubt that oxytocin injected into the brain can reduce food intake in rodent models (4) and the activity in oxytocin neurons, in either or both the paraventricular (Oxt^PVH^) and supraoptic (Oxt^SON^) hypothalamic nuclei, has been recorded after fast-induced re-feeding (5, 6) or a sucrose meal (5, 7, 8), following gastric distension (9) or by administration of metabolic hormones (9–17). The first germline knock outs of either the oxytocin gene (*Oxt*), or its receptor (*Oxtr*), failed to produce a notable phenotype (18, 19). By contrast, conditional knock down of *Oxt* selectively in the adult PVH, or *Oxtr* in the adult posterior hypothalamus, increases food intake and body weight (20). Furthermore, the ablation of adult Oxt^PVH^ neurons with diptheria toxin also affects body weight; though whether this is dependent on hyperphagia (21) or decreased sympathetic input to adipose tissues (22, 23) is currently unresolved. The metabolic effects of stimulating oxytocin neurons directly are also debated. Acute activation of adult Oxt^PVH^ neurons using either chemo- or opto-genetics did not reduce fast-induced re-feeding (24, 25), though the stimulation of Oxt^PVH^ fibers in the rostral medullary raphe region (rMR) in the brainstem increased brown adipose-dependent thermogenesis (26).

The role of oxytocin and oxytocin neurons may be subtle and/or modulatory, so perhaps gross changes in energy intake or expenditure might not be expected by manipulating a very small population of neurons in isolation. For example, oxytocin modulates the effect of other satiety factors through descending PVH projections to the dorsal vagal complex (10, 16, 21, 27–31). Oxytocin receptors are present on first-order sensory neurons of the vagus (21, 32–34), as well as on integratory neurons in the nucleus of the tractus solitarius (NTS) (21, 35). Furthermore, oxytocin receptor antagonist administered into the fourth ventricle increases meal size, while lesions of Oxtr-containing hindbrain neurons reduce responses to satiety signals (21, 35, 36). Leptin-sensitive Oxt^PVH^ neurons that project to the brainstem can decrease meal size by increasing the sensitivity of NTS neurons to other meal-related signals, such as cholecystokinin (CCK) (12, 21, 35). It is, therefore, interesting that diet-induced obese mice reduced food intake when Oxt^PVH^ neurons were activated at the same time as CCK was administered, but not if each stimulus was presented alone (21). Finally, there are descending Oxt^PVH^ neuronal projections which activate parasympathetic motor neurons in the dorsal motor nucleus of the vagus (DMV) to reduce gastric motility via a non-adrenergic, non-cholinergic (NANC) pathway (31, 37–40). A slowing of gastric emptying may be sufficient to enhance satiation and reduce overall food intake.

Descending Oxt^PVH^ neuronal projections also affect energy expenditure. Fourth ventricular injection of oxytocin increases brown adipose-mediated thermogenesis (41–43). As already mentioned, Fukushima and colleagues have generated an adeno-associated virus (AAV) that allows strong expression of channel rhodopsin (ChR2) from the rat *Oxt* promoter (26). This allowed effective stimulation of Oxt^PVH^ fibers in the brainstem raphe region that contains presympathetic serotonergic neurons. Selective stimulation of these fibers increased glutamatergic input to the rostral medullary raphe to increase sympathetic nerve outflow and energy expenditure through thermogenesis.

In addition to modulating brainstem signaling, oxytocin may also interact with forebrain structures to affect other metabolic parameters, such as the motivation to consume different macronutrients. In food-choice experimental paradigms, *Oxt* knock-out mice initiate consumption of carbohydrate drinks and not lipid emulsions (44, 45). This, coupled with the findings that high-sugar food activates Oxt^SON^ neurons (46) and administration of a brain-penetrant oxytocin receptor antagonist increases glucose ingestion (47), suggests that oxytocin neurons may respond to sugar to reduce subsequent carbohydrate consumption. The circuitry required for an effect of the endogenous oxytocin system has not been determined, but Oxt^PVH^ neurons do project to regions of the brain involved in reward, including the ventral tegmental area (VTA) and nucleus accumbens (NAc) (48–50). Indeed, injections of oxytocin into the VTA or NAc reduce sucrose intake, an effect which can be blocked by co-administration of selective receptor antagonists (51–53).

Given the extensive literature regarding the actions of endogenous oxytocin signaling on food intake, it is perhaps surprising that chemogenetic and optogenetic techniques to drive oxytocin neurons failed to induce an anorectic effect in chow-fed mice (21, 24, 25). Therefore, we decided to revisit the use of chemogenetic manipulation and found that by selectively stimulating Oxt^PVH^ neurons we could reduce normal, night-feeding in chow-fed mice. Chemogenetic inhibition of Oxt^PVH^ neurons only partially reversed the reduction in food intake caused by the anorectic peptide, CCK. Finally, noting the involvement of oxytocin on sugar intake (44–47), and the growing evidence for a role of the bed nucleus of the stria terminalis (BNST) in appetitive behaviors (54), we used optogenetic manipulation to investigate a potential link. An oxytocinergic projection from the PVH to the BNST has been described anatomically in the rat (50, 55, 56). And, although it has been proposed that oxytocin in the BNST may have a role in maternal and other social behaviors (55, 57–61), this pathway has not been implicated previously in the consummatory actions of oxytocin. Here we discovered that the projection of Oxt^PVH^ neurons to the BNST reduces sucrose intake when stimulated.

## Materials and methods

### Animals

All animals were maintained in constant environmental conditions within the University of Manchester Biological Services Facility. Animals were housed in individually ventilated cages, maintained at 21 ± 2°C, 45 ± 10% humidity, under a 12 hr:12 hr light:dark cycle (lights on 06:30 GMT). Standard laboratory chow (#801151 RM1-P; Special Diet Services, Witham, Essex, UK) and Hydropac^®^ pouch water (Avidity Science, Long Crendon, Bucks, UK) were available *ad libitum* unless otherwise stated.

Oxytocin-Ires-Cre mice (*Oxt* ^Cre^; B6;129S-Oxttm1.1(cre)Dolsn/J; RRID: IMSR_JAX:024234) were initially purchased from The Jackson Laboratory (JAX Stock #024234; Bar Harbor, Maine, USA) and subsequently bred in house by crossing with C57Bl/6J (Charles River, Margate, Kent, UK). *Oxtr* ^Cre^ embryos (Tg(Oxtr-cre)ON66Gsat/Mmucd; RRID: MMRRC_036545-UCD) were purchased from the Mutant Mouse Resource & Research Centre (MMRRC supported by NIH, USA; Stock # 036545-UCD) and re-derived by the University of Manchester Genome Editing Unit. *Oxtr* ^Cre^ mice were subsequently bred in house by crossing with Swiss Webster mice (Envigo, Belston, Kent, UK) or R26R-EYFP reporter mice (*Oxtr* ^Cre:eYFP^; B6.129X1-Gt(ROSA)26Sortm1(EYFP)Cos/J; RRID: IMSR_JAX:006148; JAX Stock #006148).

All procedures were licensed under the United Kingdom Animals (Scientific Procedures) Act 1986 and approved by local ethical review.

### Surgery: intracranial injection, optic fiber implantation and telemeter implantation

All animals were anaesthetized with 2-3% isoflurane in oxygen and administered 0.1 mg/kg buprenorphine subcutaneously (SC) before the start of surgery. For intracranial injection/optic fiber implantation, animals were placed in a stereotaxic frame, the skull was exposed and a small hole drilled above each injection/implantation site. All co-ordinates were determined using the Mouse Brain Atlas (Paxinos & Franklin, 2001). Viral vectors were injected bilaterally (-1.0 mm A/P, ± 0.3 mm M/L; - 5.0 mm D/V from bregma; four 40 nl injections of virus, i.e. 160 nl in total into each side of the PVH) using a Nanoject II automatic nanoliter injector fitted with a pulled glass micropipette (Drummond Scientific Company, Broomall, PA, USA).

For stimulatory chemogenetic experiments, male *Oxt* ^Cre^, aged 7-9 weeks, were injected into the PVH with AAV8-hSyn-DIO-hM3Dq-mCherry (titer: 5.9 x 10^12^ gc/ml; University North Carolina [UNC] Vector Core, Chapel Hill, NC, USA). To permit temperature recording, animals were also implanted with telemetry devices during the same surgery as intracranial injection. Once stereotaxic surgery was complete, animals were transferred onto a nose cone providing 2-3% isoflurane in oxygen and rotated into the supine position. A lateral incision was made in the abdomen and a remote telemetry device (DataScience International [DSI], Minneapolis, USA) was implanted inside the peritoneal cavity. For inhibitory chemogenetic experiments, male *Oxt* ^Cre^ mice, aged 7–10 weeks, were bilaterally injected into the PVH with AAV8-hSyn-DIO-hM4Di-mCherry (titer: 4.4 x 10^12^ gc/ml; UNC Vector Core).

For anterograde tracing and optogenetic experiments, male *Oxt* ^Cre^ mice, aged 10–15 weeks, were injected unilaterally into the PVH with AAV2-EF1a-DIO-ChR2(h134r)-mCherry (titre: 1.5 x 10^12^ gc/ml; UNC Vector Core). In the same surgery, animals destined for *in vivo* optogenetic studies were also implanted with an optic fiber (200 μm diameter, 0.39 NA, #CFML12U; Thorlabs LTD -20; Ely, Cambridgeshire, UK) above the BNST (+0.1 mm A/P; +1.0 mm M/L; -4.4 mm D/V from bregma) ipsilateral to intracranial injection. The optic fiber was held in place using dental cement (Simplex Rapid Powder, Kemdent, Swindon, UK; Methyl Methacrylate, Metrodent, Huddersfield, UK) and a small screw affixed into the right parietal plate.

At the end of each surgery, all wounds were sutured, animals were provided post-operative care and left to recover for at least 2 weeks before experimentation.

### Drugs

Cholecystokinin (CCK octapeptide, sulfated; #1166; Tocris Bioscience, Abingdon, Oxfordshire, UK) was dissolved in sterile saline for injection (B.Braun, Melsungen, Germany) and administered via intraperitoneal (IP) injection at 6 μg/4 ml/kg body weight. Clozapine-N-oxide (CNO; #4936; Tocris Bioscience) was dissolved in sterile saline for injection (B.Braun) and administered via IP injection at 1 mg/4 ml/kg body weight. Lithium chloride (#L9650; Sigma-Aldrich, Poole, Dorset, UK) was dissolved in sterile water for injection (Fannin, Nicosia, Greece) and administered via IP injection at 128 mg/10 ml/kg body weight. Intralipid (#I141, Sigma) was gavaged neat in a volume of 0.3 ml per mouse.

### Chemogenetic feeding studies

All chemogenetic feeding studies were performed in a crossover manner. Animals were acclimated to single housing and handling for at least one week prior to the start of the experiment. Following chemogenetic activation of Oxt^PVH^ neurons, nocturnal food intake was measured. On the day of the study, food was removed from the animals for 2 hr before onset of the dark phase. Mice were administered either vehicle or CNO (1 mg/kg, IP) 15 min before lights out and standard laboratory chow was returned straight away. Food intake was measured 1 hr, 2 hr and 14 hr after drug administration. Following a one-week washout period, the experiment was repeated such that each mouse received the opposite drug treatment. Drug treatments were counterbalanced across different experimental days.

For the chemogenetic inhibition study, animals were fasted overnight in their home cage. The following day, vehicle or CNO (1 mg/kg, IP) was administered at ZT + 3.5 hr. Thirty minutes later (ZT + 4 hr), each mouse received another IP injection of either vehicle or CCK and standard laboratory chow was returned. Food intake was measured 1 hr, 2 hr and 4 hr after injection of CCK. Following a one-week washout period, the experiment was repeated such that each mouse received the opposite treatment of vehicle or CNO but retained their saline or CCK grouping. All drug treatments were counterbalanced across different experiment days.

### Metabolic phenotyping

Mice were singly housed for at least one week before being placed into indirect calorimetric cages. Mice were put in individual cages in the Comprehensive Laboratory Animal Monitoring System (CLAMS) (Columbus Instruments; Columbus, OH, USA) to acclimate for a minimum of three days before the start of the study. Oxygen consumption (VO_2_ ml/hr) and carbon dioxide production (VCO_2_ ml/hr) were sampled every 10 min using Oxymax^®^ software (Columbus Instruments). VO_2_ ml/hr and VCO_2_ ml/hr were used to calculate the RER (RER = VCO_2_/VO_2_). On study days mice were injected IP with either saline or CNO (1 mg/kg) in the daytime (ZT + 3 hr). Mice were left to recover for at least one week and the study was repeated in a crossover design. Whilst mice were in indirect calorimetric cages, environmental enrichment was limited to bedding material. During this time all mice had *ad libitum* access to food and water. Data was averaged over one hour pre- and post-injection and presented as mean ± SEM.

### Temperature measurements and activity

Cages were placed on PhysioTel^®^ RPC-1 Receiver pads (DSI) during single housing acclimatization. Receiver pads were connected to a DSI Data Exchange Matrix, which was connected to a PC running Dataquest A.R.T. acquisition and analysis software (DSI) used to record core-body temperature (°C) and activity (AU) measurements every 5 minutes. On study days mice were injected IP with either saline or CNO (1 mg/kg) in the daytime. Mice were left to recover for at least one week and the study was repeated in a crossover design. Data was averaged over one hour pre- and post-injection and presented as mean ± SEM.

### Gastric emptying

Gastric emptying was assessed by measuring the stomach contents following oral gavage of a semi-liquid meal (6% w/v casein (#829855; SDS), 3% w/v cornstarch (#S4126; Sigma), 4% v/v Intralipid (#I141, Sigma), 3% w/v methyl cellulose (#M7140; Sigma), 3% w/v sucrose (#S0839; Sigma)); all dissolved in sterile pouch water). The day before the study, *Oxt* ^Cre:hM3Dq^ animals were fasted into clean cages, devoid of any food residue, commencing at ZT + 9 hr. Following a 24 hr fast, each mouse received an IP injection of either vehicle or CNO (1 mg/kg) and 30 min later was orally gavaged 0.5 ml semi-liquid meal. At this point, drinking water was withdrawn. Forty-five min after oral gavage, each mouse was culled by decapitation and the stomach rapidly dissected by excising immediately proximal and distal to the esophageal and pyloric sphincters, respectively. The full stomach was weighed. The stomach was then cut along the greater curvature and contents were gently removed. The gastric muscle and mucosa were briefly rinsed in PBS and patted dry. The empty stomach was weighed again so gastric contents could be calculated. To check for transfection of Oxt^PVH^ neurons with AAV8-DIO-hM3Dq-mCherry, brains were also collected and drop-fixed in 4% PFA for two days at 4°C, before being cryoprotected in 30% sucrose and processed for immunohistochemistry as outlined below.

### Optogenetic feeding studies

Animals were optogenetically stimulated via previously implanted fiber optic cannula connected to a PlexBright™ Optogenetic Stimulator System (Plexon Inc; Dallas, TX, USA). Briefly, high durability fiber optic cables (200 μm, 0.52 NA; PlexBright, Plexon), connected to an LED commutator (PlexBright, Plexon), were firmly attached to indwelling cannula using ceramic mating sleeves; (#ADAL1; Thorlabs). Photostimulation was programmed using pulse generator software (Radiant V2, Plexon) that controlled a blue LED (465 nm; PlexBright, Plexon) via a four-channel optogenetic LED Controller (PlexBright, Plexon).

Following stereotaxic surgery and recovery, *ad libitum* fed mice were habituated to time-restricted access to sucrose pellets (#1811155-5TUL; Test Diet, Richmond, IN, USA), which were presented in a shallow, ceramic food bowl for 30 min per day, for one week before further experimentation. The animals were then acclimated to handling and tethering within open-topped cages for several 30 min training sessions. Sucrose pellets were only available for the duration of each training session and sucrose intake was measured until stable. On test day 1, animals were tethered for 30 min in the absence of optogenetic stimulation and sucrose intake was measured. On test day 2, animals were tethered and stimulated continuously for 30 min (10 msec pulse width at 20 Hz) before sucrose intake was measured. On test day 3, animals were tethered for 30 min without optogenetic stimulation and sucrose intake was measured. Only animals that completed both optogenetic stimulation and non-stimulation trials (n = 5) were included in the final analysis. One mouse was omitted from the post-stimulation trial due to loss of the indwelling optic fiber. Each trial was performed during the light phase (commencing ZT + 5 hr), using animals that had *ad libitum* access to standard chow up until the start of each trial. During each trial, only sucrose pellets were available. After optogenetic experiments were completed, the mice were culled by transcardial perfusion and brains collected for immunohistochemistry.

### Tissue processing and immunohistochemistry

Before brains were collected, different cohorts of mice were treated as follows: Group-housed male and female *Oxtr* ^Cre:eYFP^ mice, and singly-housed male *Oxt* ^Cre^ mice, previously injected with AAV-DIO-hM4Di-mCherry or AAV-DIO-ChR2(h134r)-mCherry, were perfused with no other intervention. Following an overnight fast, singly housed male *Oxt* ^Cre^ mice previously injected with AAV-DIO-hM3Dq-mCherry were administered vehicle or CNO (1 mg/kg, IP) and perfused 2 hr later. To assess Oxt^PVH^ neuronal activation, wild-type male littermates from the *Oxt* ^Cre^ colony, were singly housed, fasted overnight and then either: perfused with no other intervention; refed standard chow for 2 hr before culling; or administered CCK (6 μg/kg, IP), LiCl (128 mg/kg, IP) or 20% Intralipid (0.3 ml/mouse, oral gavage) and then culled 90 min later.

For all histology, animals were deeply anaesthetized (5% isoflurane in 100% oxygen) and transcardially perfused with heparinized saline (20 kU/L, Sigma) followed by 4% paraformaldehyde (made up in 0.1 M phosphate buffer, PB; both Sigma). Brains were dissected and post-fixed in 4% PFA overnight at 4°C before equilibrating in 30% sucrose (made up in 0.1 M PB) at 4°C. Coronal brain sections, 30 μm thick, were cut using a freezing sledge microtome (Bright 8000; Bright Instruments, Huntingdon, Cambridgeshire, UK) and collected into 3 or 4 sets (spaced 90 μm or 120 μm apart, respectively). Sections were either frozen in cryopreservant solution at -20°C or processed immediately for immunohistochemistry.

Free-floating sections were washed with 0.2% Triton X-100 (PB-T; made up in 0.1 M PB) and then blocked in 5% normal serum (Sigma; made up in PB-T) for 1 hr at room temperature. Following blocking, sections were immediately incubated in primary antibody (made up in 1% normal serum in PB-T) for 20 min at room temperature and overnight at 4°C. The following day, sections were washed in PB-T and then incubated in secondary antibody (made up in 5% normal serum in PB-T) for 2 hr at room temperature. Finally, sections were rinsed in 0.1 M PB followed by distilled water, mounted onto glass microscopy slides, air-dried overnight and coverslipped with Prolong Gold (Molecular Probes, Eugene, OR, USA).

Primary antibodies used were: Rabbit anti-cFos (#sc52; Santa Cruz, Dallas, TX, USA); Rabbit anti-DsRed (#632496; Takara Bio Europe, Saint-Germain-en-Laye, France); Chicken anti-GFP (#Ab13970; AbCam, Cambridge, Cambridgeshire, UK); Guinea-Pig anti-Oxytocin (#T-5021; PenLabs, San Carlos, CA, USA), Guinea Pig-anti-VGLUT2 (#AB2251; Millipore, Temecula, CA, USA). Secondary antibodies used were: Donkey anti-Chicken Alexa Fluor 488 (#703-545-155; Jackson ImmunoResearch (JIR) Europe Ltd, Ely, Cambridgshire, UK); Donkey anti-Goat Alexa Fluor 594 (#705-585-147; JIR); Donkey anti-Rabbit Alexa Fluor 488 (#711-545-152; JIR); Donkey anti-Rabbit Alexa Fluor 594 (#711-585-152; JIR); Donkey anti-Rabbit Dylight 405 (#;711-475-152; JIR); Goat anti-Chicken (#A11039; Molecular Probes); Goat anti-Guinea Pig Alexa Fluor 488 (#A11073; Molecular Probes); Goat anti-Rabbit Alexa Fluor 594 (#A11008; Molecular Probes).

Images were acquired with either: an Olympus BX51 upright microscope (using 5x, 10x, 20x or 40x objectives), fitted with a Coolsnap ES Camera and captured via Metavue Software (Molecular Devices, Sunnyvale, CA, USA); or a 3D-Histech Pannoramic-250 microscope slide-scanner (using a 20x objective) and snapshots of slide scans were captured using the SlideViewer software (3D-Histech, Budapest, Hungary). Specific band-pass filter sets for DAPI, FITC and TexasRed were used to prevent bleed-through. All images were processed and analyzed using FIJI Image J (http://imagej.net/Fiji/Downloads). For neuronal quantification, 3-6 sections per mouse spanning the rostro-caudal extent of the PVH were counted manually in FIJI and averaged to give one count per mouse. Each count per mouse was averaged with values from other animals receiving the same treatment to create a group mean ± SEM. All counts were performed blind.

### Statistical analysis

All data are presented as mean ± SEM. Statistical significance was calculated using GraphPad Prism version 9.2.0 (https://www.graphpad.com). Data were tested using either paired *t*-test, unpaired *t*-test or repeated measures two-way ANOVA as indicated in the figure legends. Where applicable, ANOVA tests were followed with either Šídák’s or Tukey’s multiple comparisons *post hoc* test. Correlation between % Oxt^PVH^ transfection and % reduction in night-time feeding ((intake_CNO_ - intake_VEH_)/intake_VEH_) * 100) was tested by calculating Pearson’s correlation co-efficient. The threshold for statistical significance was set at P < 0.05. Where P < 0.1, the value is stated.

## Results

**Figure 1:**
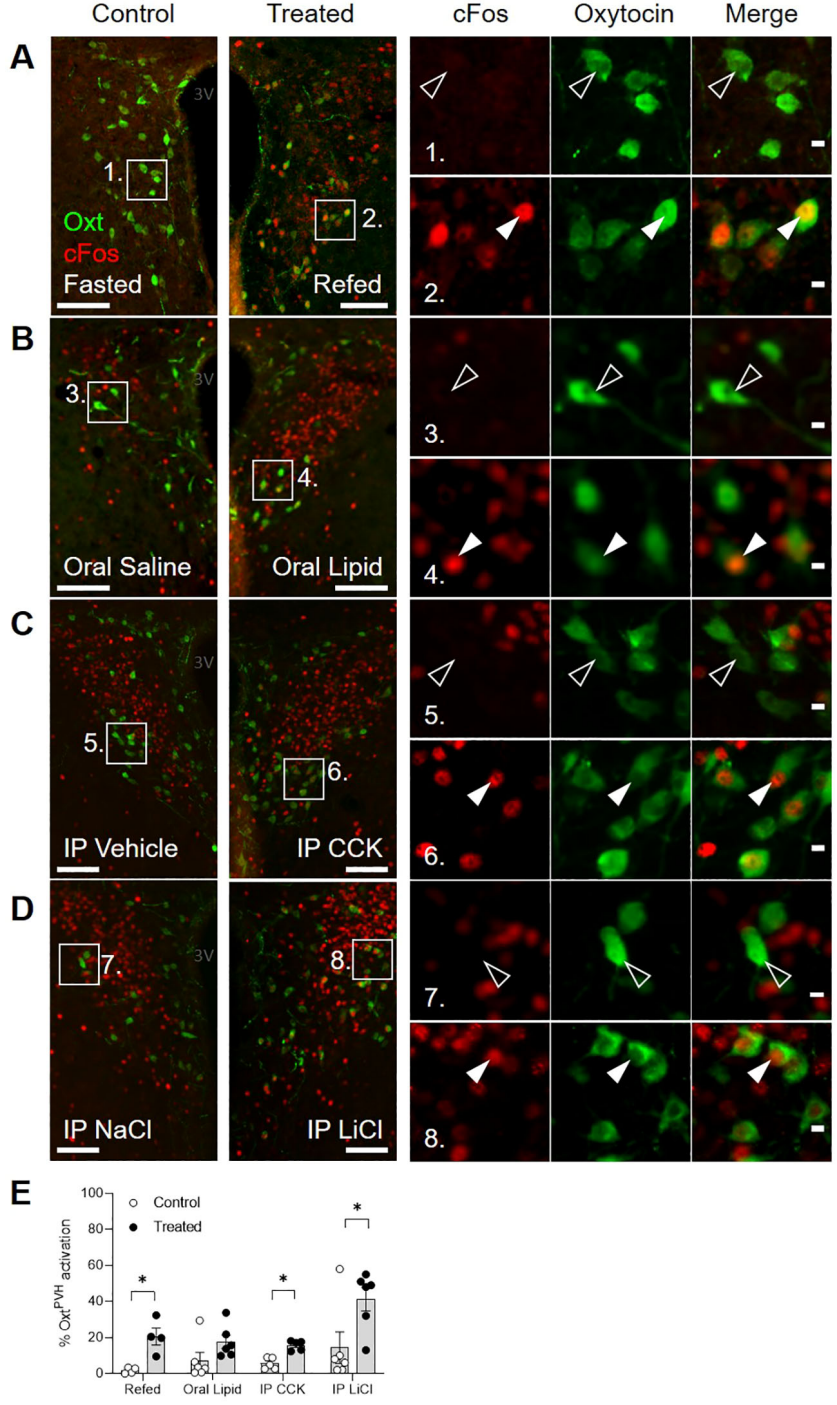
Dual-label immunohistochemical staining for oxytocin (green) and cFos (red) in the PVH following a variety of anorectic stimuli in male mice. Example photomicrographs demonstrating the induction of cFos following **(A)** an overnight fast and re-feeding following an overnight fast; **(B)** oral gavage of saline control or Intralipid; **(C)** IP injection of vehicle or cholecystokinin (CCK); **(D)** IP injection of saline control or lithium chloride (LiCl). White arrow heads indicate activated Oxt^PVH^ neurons that co-express cFos.. Empty arrow heads indicate non-activated Oxt^PVH^ neurons. **(E)** Results from individual experiments showing the percentage of Oxt^PVH^ neurons expressing cFos. Data presented as mean ± SEM. * P < 0.05, unpaired *t*-test (n = 4-7 per group). 3V: third ventricle. Scale bars in **(A-D)** 100 μm. Scale bars in insets 10 μm.

First, we used dual immunostaining for native oxytocin and nuclear cFos (the protein product of the immediate-early gene, c-*fos*) as a cellular activity marker to measure the activation of Oxt^PVH^ neurons by a range of metabolic stimuli, including fast-induced re-feeding and oral lipid gavage, both of which can be considered as physiological stimuli, as well as systemic administration of CCK or lithium chloride (LiCl; **Figure 1**; **Supplementary Table S1**). cFos activity mapping in the PVH is complicated by the fact that the neurons (including both oxytocin and non-oxytocin neurons) are highly susceptible to stress (13). Thus, despite extensive acclimation to handling, control mice given either oral gavage or IP injection of control saline displayed significant cFos in Oxt^PVH^ neurons (**Figure 1E**). This compares with low activity in the Oxt^PVH^ neurons of fasted mice, which are then significantly activated when food is returned (**Figure 1A**). Hume and co-workers have previously demonstrated that high sucrose, but not high-fat diet activates oxytocin neurons (46). Here, we found that oral gavage of lipid increased cFos in Oxt^PVH^ neurons compared with saline gavage, but this did not reach statistical significance (**Figure 1B**). The detection of lipid in the duodenum leads to the secretion of CCK from the gut, which activates vagal afferents. The signal is relayed by prolactin-releasing hormone neurons in the NTS (62, 63), which project up to Oxt^PVH^ neurons (6). While bolus injection of exogenous CCK mimics satiety signaling by the gut, it is a very mixed stimulus including elements of stress and aversion (64). Thus, a single IP injection of CCK, at a dose which we have found is the minimum that causes a reliable reduction in food intake, induced cFos in Oxt^PVH^ neurons (**Figure 1C**). Finally, we found that the nausea-inducing drug LiCl caused a significant activation of Oxt^PVH^ neurons (**Figure 1D**). The pathways from the periphery to the hypothalamus, and specifically to oxytocin neurons, that mediate the effects of LiCl have not been explored fully but are likely to include both vagal and spinal afferents and preproglucagon neurons in the NTS (65). Each of these stimuli, chosen to demonstrate the activation of Oxt^PVH^ neurons, also lead to a decrease in subsequent food intake.

**Figure 2:**
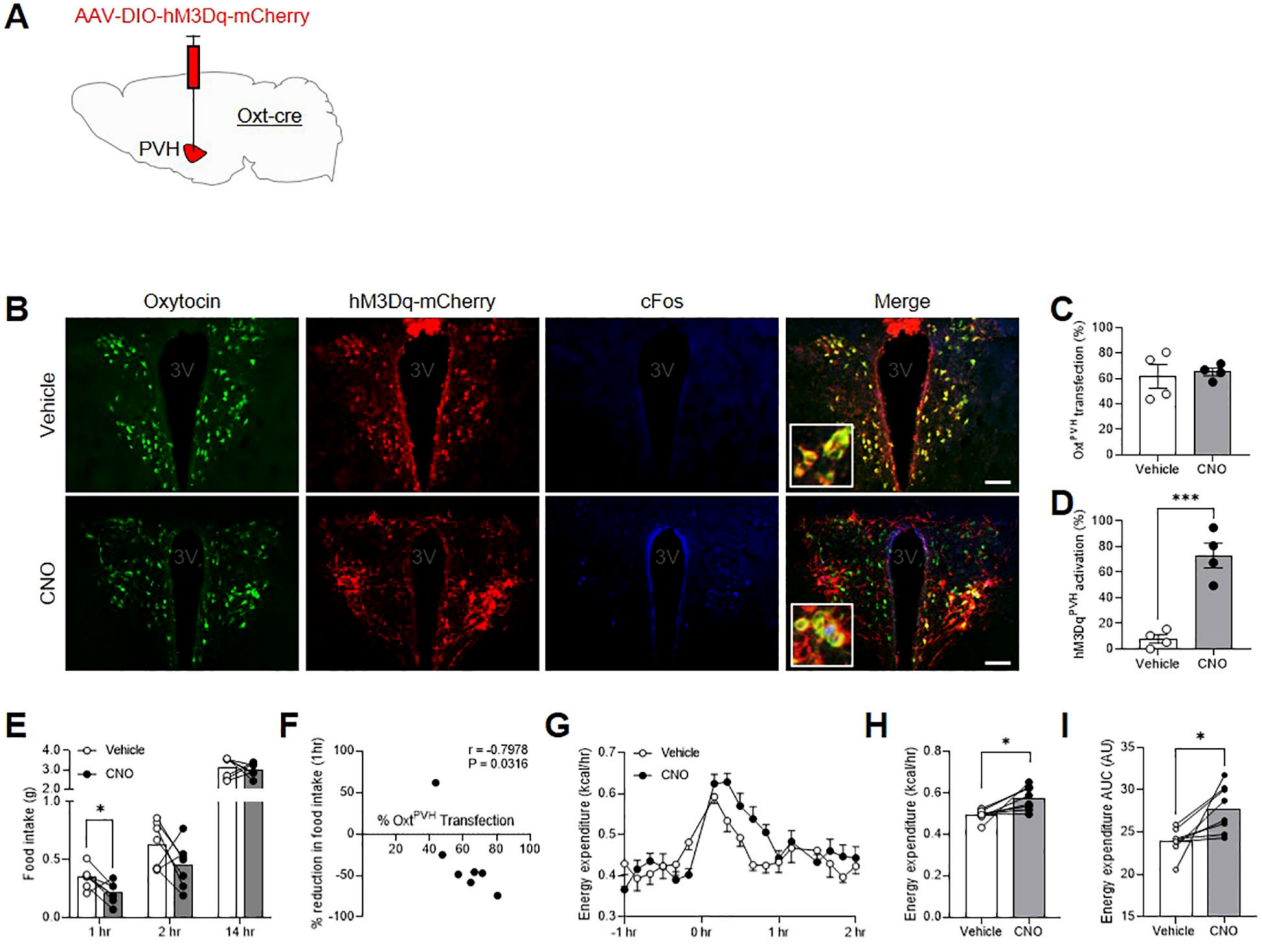
**(A)** Schematic showing injection of AAV-DIO-hM3Dq-mCherry into the PVH of male *Oxt* ^Cre^ mice. **(B)** Triple-label immunohistochemistry for native oxytocin (green), mCherry (red) and cFos (blue) following IP injection of either vehicle or clozapine-N-oxide (CNO). Inset: digital zoom. Scale bar 100 μm. **(C)** Quantification of Oxt^PVH^ neurons transfected with AAV-DIO-hM3Dq-mCherry and **(D)** activation of hM3Dq^PVH^ neurons (as determined by co-expression of cFos) following injection of either vehicle or CNO. Data presented as mean ± SEM. *** P < 0.001, unpaired *t*-test (n = 4 per group). **(E)** Night-time food intake following IP injection of vehicle or CNO in *Oxt* ^Cre:hM3Dq^ mice. * P < 0.05, repeated measures two-way ANOVA with Sidak’s multiple comparisons *post hoc* analysis (n = 7). **(F)** Correlation between Oxt^PVH^ transfection and reduction in 1 hr food intake elicited by CNO. Each data point represents one animal. r = Pearson’s correlation co-efficient. **(G)** Energy expenditure following IP injection of vehicle or CNO in *Oxt* ^Cre:hM3Dq^ mice. Data presented as mean ± SEM, repeated measures two-way ANOVA with Sidak’s multiple comparisons *post hoc* test (n = 8). **(H)** Average energy expenditure between 0 hr and 1 hr after IP injection of vehicle or CNO. * P < 0.05, paired *t*-test (n = 8). **(I)** Area under the curve (AUC) for energy expenditure between 0 hr and 1 hr. * P < 0.05, paired *t*-test (n = 8). 3V, third ventricle.

Previous studies that have used either chemogenetic or optogenetic stimulation of Oxt^PVH^ neurons did not record any effect on acute food intake (24, 25). However, both of these experiments involved mice that had been fasted and, thus, would have had a strong motivation to eat. Here we used the stimulatory designer receptor, hM3Dq, to selectively activate Oxt^PVH^ neurons by IP injection of clozapine-N-oxide (CNO) (**Figures 2A–D**). Male *Oxt* ^Cre^ mice were injected with AAV-DIO-hM3Dq-mCherry bilaterally into the PVH and, at the same time, implanted with temperature telemeters. Seven weeks after surgery, *Oxt* ^Cre:hM3Dq^ mice were injected IP with either CNO or saline vehicle, just before lights out, and food intake was measured whilst in their home cages. One week later, mice were given the opposite treatment in a crossover design. CNO caused a transient decrease in food intake during the first hour of normal nighttime feeding, which *post mortem* was shown to correlate with the number of Oxt^PVH^ neurons transfected (**Figures 2E, F**; **Supplementary Table S2**). For these and other injections, no off-target expression of the transgene was noted elsewhere in the brain, including for example in the supraoptic nucleus (**Supplementary Figure S1A**).

The same cohort of male *Oxt* ^Cre:hM3Dq^ mice was also acclimated to calorimetry cages to allow simultaneous measurement of metabolic gases, core-body temperature and activity. After three days of acclimation, the animals underwent a crossover experiment, with vehicle and CNO injections given one week apart (treatments were counterbalanced across different experimental days). CNO caused a significant increase in energy expenditure over a period of approximately one hour (**Figures 2G–I**). This coincided with an increase in core-body temperature within the same period (**Supplementary Figures S1B–D**), without affecting overall activity levels (**Supplementary Figures S1F, G**). The respiratory exchange ratio (RER) was unaffected by Oxt^PVH^ chemogenetic activation (RER_0-1hr_ mean ± SEM: vehicle: 0.9875 ± 0.0211; CNO: 1.019 ± 0.0228; P > 0.05; paired t-test, n = 8).

In a separate experiment, male *Oxt* ^Cre:hM3Dq^ mice were injected with either saline or CNO to assess the effect of Oxt^PVH^ chemogenetic activation on gastric emptying. Following a 24 hr fast, one group of *Oxt* ^Cre:hM3Dq^ animals was injected with saline and another group injected with CNO. Each animal was then orally gavaged with 0.5 ml of a semi-liquid meal and culled 45 min later. The stomachs were removed and gastric contents calculated. Oxt^PVH^ neuronal activation had no significant effect on the volume of gastric contents measured, indicating that there was no change in the rate of gastric emptying (**Supplementary Figure S1H**). Therefore, whilst others have shown that oxytocin can modify the stress-induced reduction in gastric emptying in rodents (31, 66), activation of Oxt^PVH^ neurons *per se* does not overtly alter gastric emptying under normal (non-stressful) conditions.

**Figure 3:**
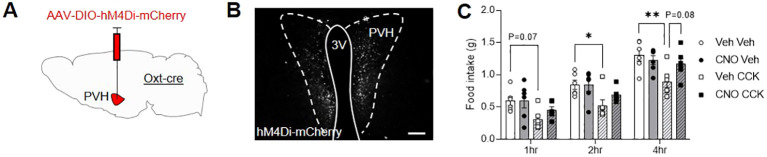
**(A)** Schematic showing injection of AAV-DIO-hM4Di-mCherry into the PVH of male *Oxt* ^Cre^ mice. **(B)** Representative photomicrograph of mCherry immunohistochemical staining in the PVH of *Oxt* ^Cre^ mice injected with AAV-DIO-hM4Di-mCherry. Scale bar 100 μm. **(C)** Fast-induced food intake in Oxt^Cre:hM4Di^ mice following an IP injection of either vehicle (circle symbols, plain bars) or CCK (square symbols, hatched bars) either with (black symbols, grey bars) or without (white symbols, white bars) concurrent chemogenetic inhibition of Oxt^PVH^ neurons. Data presented as mean ± SEM. * P < 0.05, ** P < 0.01, repeated measures two-way ANOVA with Tukey’s multiple comparisons *post hoc* test (n = 6). 3V, third ventricle; PVH, paraventricular nucleus of the hypothalamus.

Next, a group of male *Oxt* ^Cre^ mice were injected with the inhibitory designer receptor, AAV-DIO-hM4Di-mCherry, bilaterally into the PVH (**Figure 3A**; **Supplementary Table S2**) and allowed to recover. Two weeks after surgery, following an overnight fast, *Oxt* ^Cre:hM4Di^ mice were pre-injected with either vehicle or CNO IP. Thirty minutes later, the animals were then given another IP injection of either saline-vehicle or CCK and daytime fast-induced food intake was measured. The following week, in a partial-crossover design, the pre-injection treatments (vehicle and CNO) were swapped; whereas saline-vehicle and CCK treated-animals remained the same. Bilateral transfection *Oxt* ^Cre:hM4Di^ neurons in the PVH was validated *post mortem* by mCherry immunohistochemistry (**Figure 3B**). CCK caused a significant decrease in food intake, which was attenuated by prior chemogenetic inactivation of Oxt^PVH^ neurons (**Figure 3C**). This is the first direct evidence that acute activity of Oxt^PVH^ neurons is required for the anorectic effects of exogenous CCK, though corroborates the complete blockade of CCK-induced anorexia recently reported after the ablation of oxytocin neurons with diptheria toxin (21).

**Figure 4:**
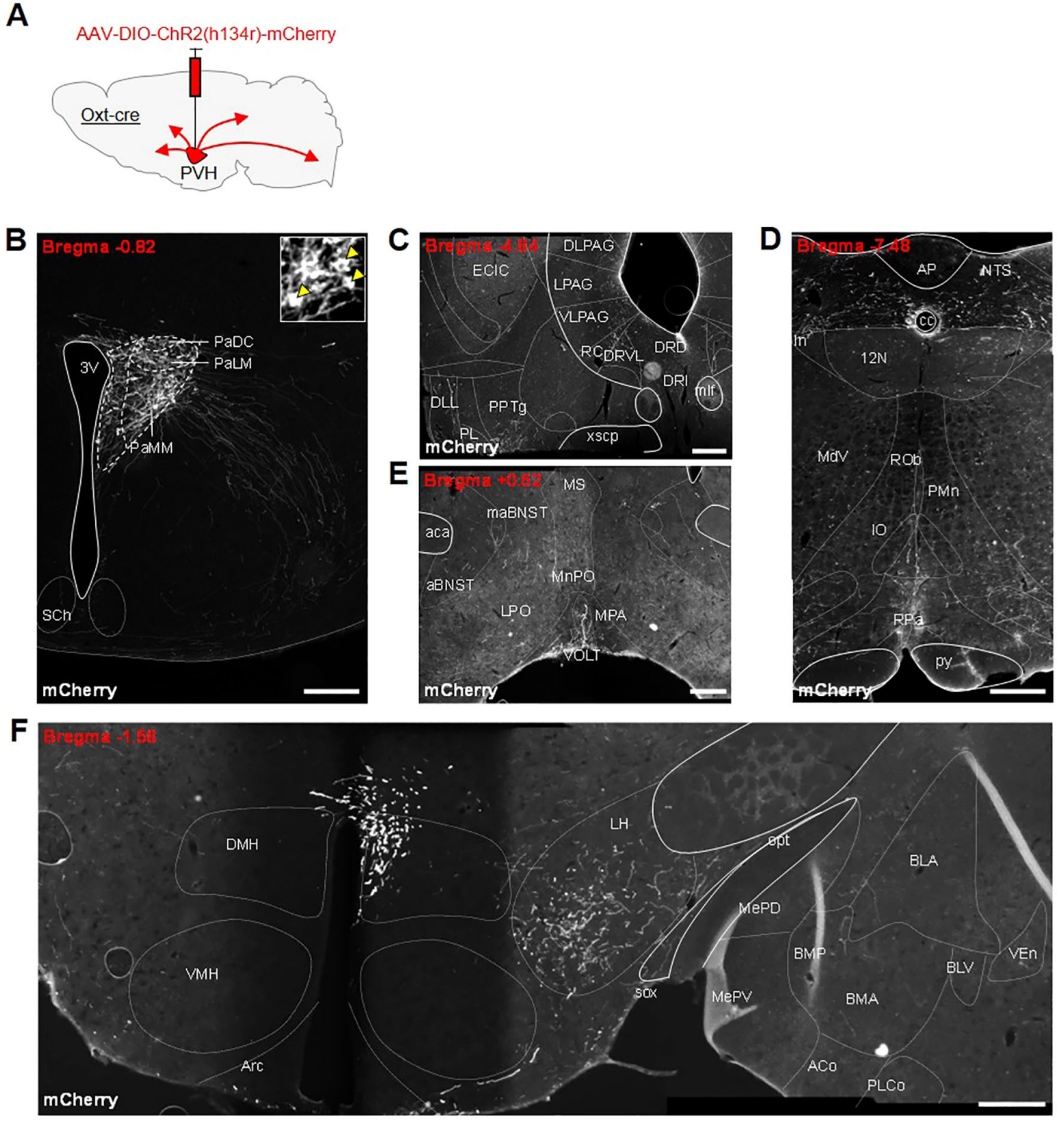
**(A)** Schematic showing injection of AAV-DIO-ChR2(h134r)-mCherry into the PVH of male *Oxt* ^Cre^ mice. All photomicrographs depict immunohistochemical staining of mCherry. **(B)** Unilateral transfection in the PVH. Inset: digital zoom depicting cell bodies (indicated by yellow arrowheads). Anterogradely-labelled neuronal fibers in the **(C)** tegmentum, dorsal raphe and periaqueductal grey (PAG) region, **(D)** nucleus of the solitary tract (NTS) and raphe pallidus (RPa) in the hindbrain, **(E)** anterior bed nucleus stria terminalis (aBNST) and preoptic area, **(F)** lateral hypothalamus and amygdala. 12N, hypoglossal nucleus; 3V, third ventricle; ACo, anterior cortical amygdaloid nucleus; AP, area postrema; Arc, arcuate nucleus; BLA, basolateral amygdala, anterior part; BLV, basolateral amygdaloid nucleus, ventral part; BMA, basomedial amygdaloid nucleus, anterior part; BMP, basomedial amygdaloid nucleus, posterior part; cc, central canal; DLL, dorsal nucleus of the lateral lemniscus; DLPAG, dorsolateral periaqueductal grey; DMH, dorsomedial nucleus of the hypothalamus; DRD, dorsal raphe nucleus, dorsal part; DRI, dorsal raphe nucleus, interfascicular part; DRVL, dorsal raphe nucleus, ventrolateral part; ECIC, external cortex of the inferior colliculus; In, intercalated nucleus of the medulla; IO, inferior olive; LH, lateral hypothalamus; LPAG, lateral periaqueductal gray; MdV, medullary reticular nucleus, ventral part; MePD, medial amygdaloid nucleus, posterodorsal part; MePV, medial amygdaloid nucleus, posteroventral part; mlf, medial longitudinal fasciculus; NTS, nucleus of the tractus solitarius; opt, optic tract; PaDC, paraventricular nucleus, dorsal cap; PaLM, paraventricular nucleus, lateral magnocellular part; PaMM, paraventricular nucleus, medial magnocellular part; PL, paralemniscal nucleus; PLCo, posterolateral cortical amygdaloid nucleus (C2); PMn, paramedian reticular nucleus; PPTg, pedunculopontine tegmental nucleus; py, pyramidal tract; RC, raphe cap; ROb, raphe obscurus nucleus; RPa, raphe pallidus nucleus; SCh, suprachiasmatic nucleus; sox, supraoptic decussation; VEn, ventral endopiriform nucleus; VLPAG, ventrolateral periaqueductal grey; VMH, ventromedial nucleus of the hypothalamus; xscp, decussation of the superior cerebellar peduncle. All scale bars 250 μm.

Following the success of other studies that have stimulated specific oxytocin pathways, we decided to look for other possible routes by which oxytocin neurons might affect feeding behavior. To allow the specific tracing of Oxt^PVH^ projections, male *Oxt* ^Cre^ mice were injected unilaterally into the PVH with an AAV-DIO-ChR2(h134r)-mCherry (**Figure 4A**). Successfully transfected Oxt^PVH^ neurons were observed three weeks later (**Figure 4B**). Channel rhodopsin (ChR2) is a light-sensitive channel that is incorporated in the cell membrane and transported through neuronal processes along with a fluorescent mCherry tag. Using AAV-DIO-ChR2(h134r)-mCherry as an anterograde tracer we observed Oxt^PVH^ fibers descending to the periaqueductal grey and tegmentum (**Figure 4C**), the NTS and the rMR (specifically the raphe pallidus and median pallidus; **Figures 4D**). Projections from oxytocin neurons to each of these regions have been noted before using different techniques (67, 68). Those to the NTS and rMR have been assigned to roles affecting ascending satiety signals (16, 21, 29–31) or thermogenesis (26). In the forebrain, fibers were present in the preoptic area, the lateral hypothalamus, the amygdala and the BNST (**Figures 4E, F**). Oxytocin acts in the preoptic area to affect parental behavior and lactation (69, 70) and in the lateral hypothalamus to modulate the activity of melanin-concentrating hormone neurons (71). Projections by oxytocin neurons to the amygdala have been implicated in a variety of behaviors, including fear and social interaction (50, 72).

**Figure 5:**
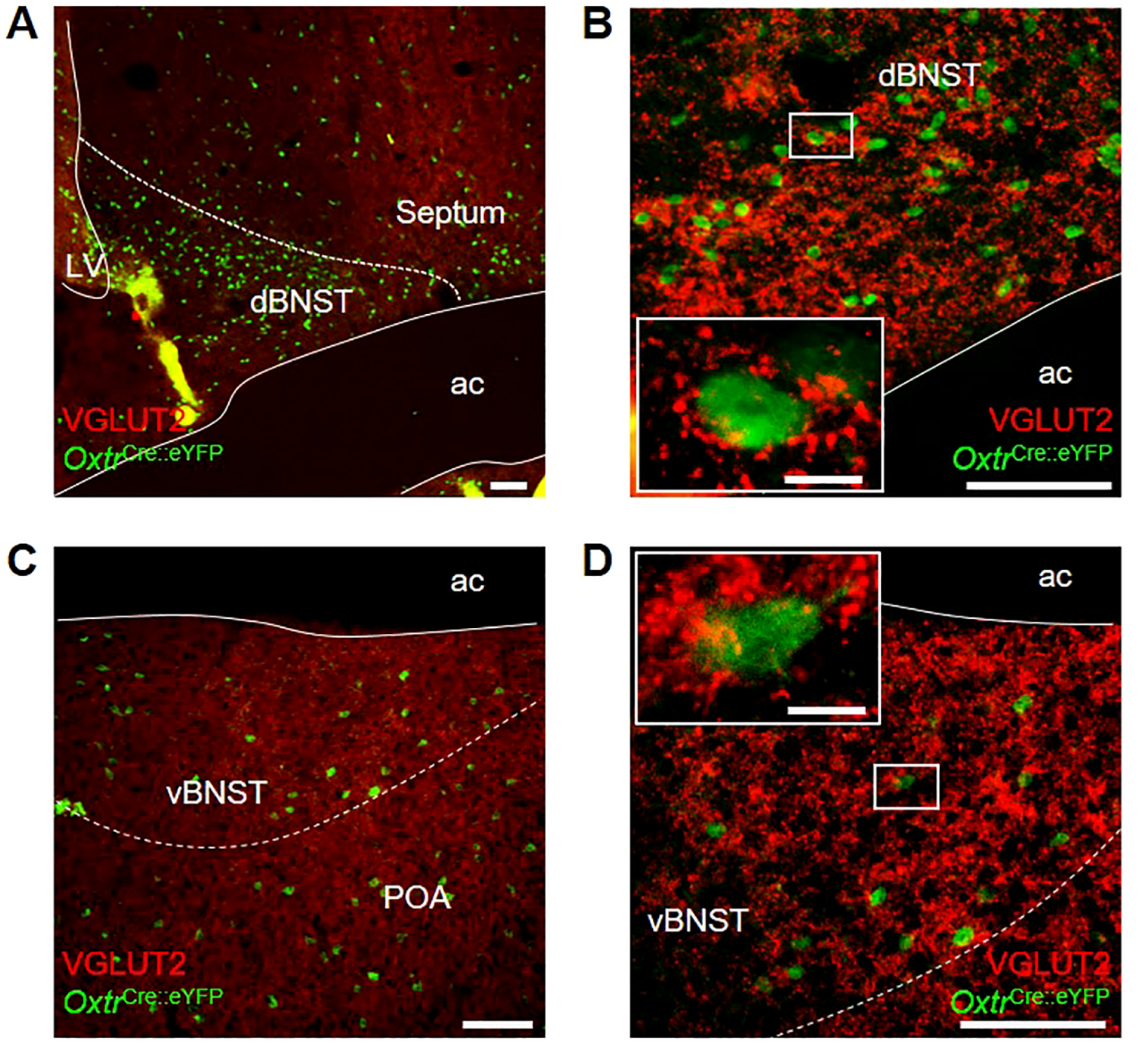
Photomicrographs of dual-label immunohistochemistry for eYFP (green) and VGLUT2 (red) in male *Oxtr* ^Cre:eYFP^ mice showing **(A)** dorsal BNST at low and **(B)** high magnification; and **(C)** ventral BNST at low and **(D)** high magnification; all scale bars 100 μm. Insets: high magnification images of single *Oxtr* ^Cre:eYFP^ cell bodies surrounded by punctate VGLUT2 staining; scale bars 10 μm. ac, anterior commissure; dBNST, dorsal bed nucleus of the stria terminalis; LV, lateral ventricle; POA, preoptic area; vBNST, ventral bed nucleus of the stria terminalis.

We were interested to note Oxt^PVH^ neuronal fibers within the BNST and followed this up to see if this region contains oxytocin receptors. We made use of an *Oxtr* ^Cre^ mouse, which was crossed with a R26R-EYFP reporter mouse. In the *Oxtr* ^Cre:eYFP^ model, oxytocin receptor neurons, identified via co-expression of eYFP, were found distributed throughout the dorsal and ventral subdivisions of the BNST of both male and female animals (**Figures 5A–D**). Oxtr^BNST^ neurons have previously been described as primarily GABAergic (61, 73, 74). Accordingly, we found that VGLUT2 staining was not co-localized with eYFP-expressing oxytocin receptor neurons. However, we did observe punctate VGLUT2 staining in apposition to eYFP-labelled cells, indicative of glutamatergic input onto Oxtr neurons within both the dorsal and ventral BNST (**Figure 5B** inset and **Figure 5D** inset).

**Figure 6:**
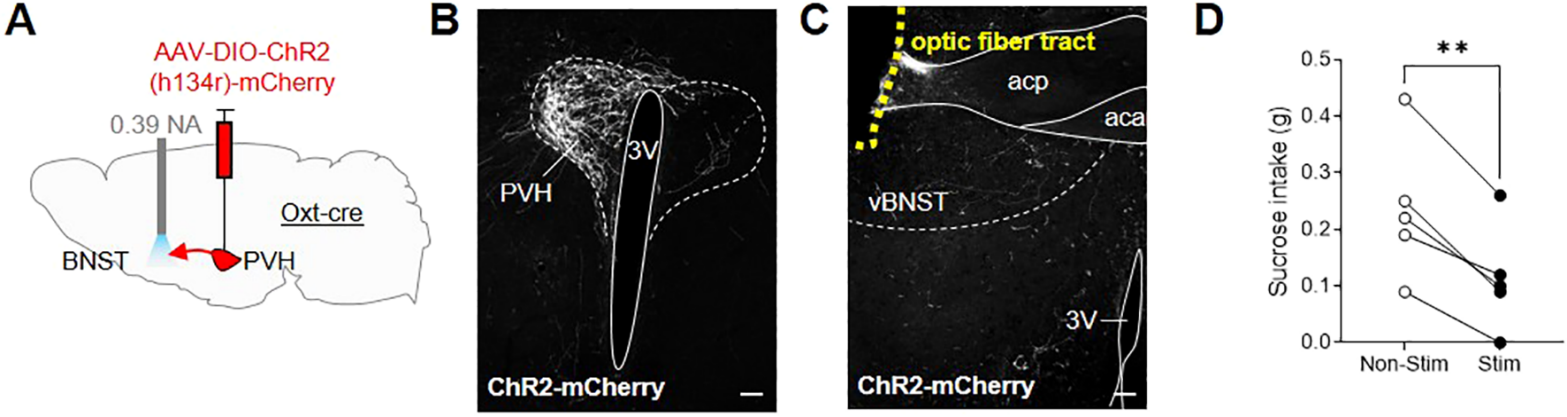
**(A)** Schematic showing injection of AAV-DIO-ChR2(h134r)-mCherry into the PVH and implant of a fiber optic cannula above the BNST in male *Oxt* ^Cre^ mice. **(B)** Representative photomicrograph of mCherry immunohistochemical staining in the PVH of *Oxt* ^Cre^ mice injected with AAV-DIO-ChR2(h134r)-mCherry. **(C)** Representative photomicrograph of Oxt^PVH^ neuronal fibers in the ventral BNST (labelled by mCherry immunohistochemical staining) and the fiber optic cannula tract above the ventral BNST (outlined with yellow dashes). **(D)** Sucrose intake in *Oxt* ^Cre^ mice following no stimulation (Non-Stim) or optogenetic stimulation (Stim) of Oxt^PVH➔BNST^ terminals. ** P < 0.01, *post hoc* paired t test (n = 5). 3V, third ventricle; aca, anterior commissure anterior part; acp, anterior commissure posterior part; PVH, paraventricular nucleus of the hypothalamus; vBNST, ventral bed nucleus of the stria terminalis. Scale bars 100 μm.

Given the extensive literature on the role of the BNST in regulating appetitive and aversive states (54) and accumulating evidence for oxytocin signaling within this region, we decided to investigate the possibility that Oxt^PVH^ projections to the BNST could modulate feeding. An oxytocinergic projection from the PVH to the BNST has been previously discussed in relation to maternal and other social behaviors (55, 57–60), but not in relation to consummatory behavior. We hypothesized that it may relate to oxytocin’s purported role in sugar consumption (44–47). To test directly an effect on sugar consumption, male *Oxt* ^Cre^ mice were injected unilaterally into the PVH with AAV-DIO-ChR2(h134r)-mCherry and, at the same time, implanted with an optic fiber above the ipsilateral BNST (**Figures 6A–C**; **Supplementary Figure S2**). After two weeks recovery, the mice were acclimated to tethering (without stimulation) via their indwelling fiber optic implants and habituated to time-restricted access to sucrose pellets within the optogenetic apparatus. Following six acclimation sessions, the animals were tethered for 30 min, in the absence of light (no stimulation), and sucrose intake was measured. The next day, the animals were tethered again and Oxt^PVH^ terminals were stimulated continuously for 30 min with 10 ms pulses of light at 20 Hz. Optogenetic stimulation of oxytocin fibers within the BNST significantly decreased sucrose intake by 58 ± 11% (n = 5), compared with non-stimulated baseline intake (**Figure 6D**). This effect was dependent on optogenetic stimulation since, in a subsequent no-stimulation trial, the mice restored their sucrose intake. Therefore, whilst oxytocin has previously been shown to influence sucrose intake (75), these data suggest that a specific projection of Oxt^PVH^ neurons to the BNST might mediate this effect, though we cannot rule out other parallel pathways.

## Discussion

Whilst the impact of experimental oxytocin application on energy balance is accepted, a role for endogenous oxytocin and neurons of the CNS that express this peptide, remains somewhat contested. In rodents, developmental knock out of oxytocin or its receptor results in late-onset obesity; whereas knock down, ablation or antagonism in adult animals produces more immediate, marked effects on energy intake, energy expenditure and body weight (2). In recent years, the development of molecular technologies has enabled increasingly sophisticated studies to interrogate the function of specific neuronal circuits in adult mice. To date, application of these tools within the oxytocin system has yielded mixed results (2). Here, we have revisited the use of chemogenetic and optogenetic techniques in different experimental paradigms and identified subtle, but significant, effects of Oxt^PVH^ neurons on different aspects of energy intake and expenditure.

We have shown that chemogenetic activation of Oxt^PVH^ neurons can affect food intake acutely, and that consumption correlates directly with the number of cells transfected (and therefore activated by CNO). The latter is a validation that the transduction with the DREADD was effective and, since the number of cells containing cFos after CNO included some variation, we were able to demonstrate that the physiological output was dependent on the level of stimulus. We purposely conducted our feeding study during the circadian dark phase and measured normal, night-time feeding of standard laboratory chow. This choice of experimental protocol was different to that employed in previous chemogenetic and optogenetic studies where semi-fasted or fully-fasted mice were used (21, 24, 25). It will be interesting to examine whether this is a defining difference. Certainly, fasted mice have a powerful motivation to eat and overcoming such an orexigenic drive can require strong opposing stimuli. An alternative hypothesis may relate to the potential interaction between oxytocin and other satiety signals. A number of studies suggest that oxytocin reduces food intake, in part, by potentiating gut-derived satiation signals; an effect that is likely mediated through descending pathways from the PVH to the dorsal vagal complex in the brainstem (10, 16, 21, 27–31). Thus, after an experimental fast, mice will have little or no food in their upper gastrointestinal tract, so such satiation signals may not be operating when the mice begin to re-feed. In support of this, recent work from Gruber et al. (21) demonstrated that chemogenetic activation of Oxt^PVH^ neurons elicits reduced consumption of a high-fat, high-sugar diet only when CCK is co-administered systemically with CNO. Therefore, whilst chemogenetic activation of Oxt^PVH^ neurons is capable of eliciting modest anorexia acutely, this effect is likely impacted by energy status and requires the integration of systemic and central satiation signals.

Collective evidence (reviewed in (2)) suggests that oxytocin regulates satiation and meal size, rather than modulating interoceptive hunger or satiety *per se*. This may explain why we detected a subtle anorectic effect within the first hour of nocturnal feeding, but not thereafter, when an increase in meal number may counteract reduced meal size. A decrease in meal size may be caused by early onset of satiation and result from decreased gastric motility and a slowing of gastric emptying (leading to the perception of fullness). Indeed, administration of oxytocin reduces gastric tone and slows gastric emptying/transit along the gastrointestinal tract in rodents (40, 76, 77). However, oxytocin can also restore gastric motility and reverse the delay in gastric emptying that is caused by stress (31, 66, 78). Although such effects may be mediated by peripheral oxytocin receptors that are expressed along the GI tract (76), oxytocin acts specifically within the brainstem dorsal vagal complex to modulate both cholinergic and NANC neural pathways that increase and decrease gastric motility, respectively (31, 40, 77, 79). However, we did not detect an effect on gastric emptying following chemogenetic activation of Oxt^PVH^ neurons as determined by crude measurement of gastric contents following oral gavage of a semi-liquid meal. In contrast, elegant work from Travagli and colleagues demonstrates that chemogenetically activating the Oxt^PVH➔NTS^ pathway increases gastric motility and reverses the delay in gastric emptying that occurs in response to stress, whereas inhibition of this projection prevents gastric adaptation to stress (31). Furthermore, activation of Oxt^PVH➔NTS^ terminals increases the firing frequency of preganglionic DMV neurons *ex vivo* and increases gastric tone and motility in anaesthetized animals. Here, we did not observe an effect of Oxt^PVH^ activation in animals devoid of an overt stressor. This fits with the findings that in naïve, non-stressed animals, oxytocin does not disinhibit DMV neurons to promote gastric motility and that only targeted microinjection of oxytocin into the dorsal vagal complex elicits gastric relaxation through the NANC pathway (40, 77). As such, our data might reflect a difference between application of exogenous oxytocin compared with the activation of central circuits downstream of Oxt^PVH^ neurons and reiterates the highly context-dependent role of oxytocin in modulating physiological processes. Whether Oxt^PVH➔NTS^ projections can potentiate vagal input within the dorsal vagal complex to augment gut-derived satiation signals in the post-prandial phase (79) and elicit subtle modulations in gastric emptying (that were below the threshold of detection in our assay, yet which drive enhanced onset of satiation), requires further investigation.

Although we have shown that artificially activating Oxt^PVH^ neurons is sufficient to reduce normal night-time feeding, this does not necessarily mean that these neurons are involved in physiological regulation. Loss of function studies are important to determine the role of an endogenous signaling pathway. Whereas developmental knock out of oxytocin has a negligible effect on food intake (19), ablation of adult oxytocin neurons or region-specific knock down of the peptide induces a marked hyperphagic phenotype (20, 21). Furthermore, acute administration of oxytocin receptor antagonists attenuates the hypophagic effects of many different anorectic signals (2). Having established that chemogenetic activation of Oxt^PVH^ neurons modulates food intake within the first hour of normal, night-time feeding, when satiation signals rapidly rise in response to food ingestion, we decided to investigate a potential role for Oxt^PVH^ neurons in mediating the effects of the satiation signal, cholecystokinin. CCK is a strong activator of oxytocin neurons in both the PVH and SON, an effect that is likely mediated via circuitry ascending from the brainstem (6, 80). To limit non-physiological effects of CCK, we chose the lowest effective anorectic dose (in our hands). This dose activated a small but significant number of Oxt^PVH^ neurons and also activated many other non-oxytocinergic neurons with the PVH. Accordingly, we found that chemogenetic inhibition of Oxt^PVH^ neurons partially attenuated the anorectic effects induced by systemic administration of CCK. Others have also shown that ablation of Oxt^PVH^ neurons using diphtheria toxin renders mice insensitive to systemic CCK (21, 23). Such varied extent of disruption could be attributed to effects of oxytocin versus co-expressed transmitters, since central administration of oxytocin antagonists also attenuates the anorectic effect of CCK (81, 82). Collectively, these data support the notion that oxytocin neurons are involved in the anorectic response to CCK.

Pharmacological treatment of rodents with oxytocin reduces food intake and body weight, but pair-feeding studies suggest there are additional effects on energy expenditure and adipose tissue lipolysis (83–87). Furthermore, late-onset or diet-induced obesity in oxytocin-deficient animals has also been attributed to deficits in energy expenditure, rather than changes in food intake (19, 22). In contrast, the chemogenetic activation of Oxt^PVH^ neurons has yielded somewhat conflicting results. In two independent experiments, Sutton et al. (24) reported that Oxt^PVH^ stimulation increased oxygen consumption in one cohort of mice, but not in another (although P = 0.07 in the “no significant effect” group). To further clarify the effect of Oxt^PVH^ neuronal activation, we used metabolic cages in combination with radiotelemetry, to measure energy expenditure, locomotor activity and body temperature concurrently. Chemogenetic activation of Oxt^PVH^ neurons caused a significant increase in energy expenditure in the first hour following administration of CNO. There was no accompanying alteration in the respiratory exchange ratio, implying that Oxt^PVH^ activation does not alter substrate utilization in *ad libitum* fed animals that are maintained at room temperature. Others have previously shown that experimental administration of oxytocin, directly into the VMH, reduces RER secondary to hypophagia (88). More recently, oxytocin released directly from sympathetic neurons has been shown to directly induce lipolysis (89). However, given that chemogenetic activation of Oxt^PVH^ neurons is unlikely to elicit hypophagia in *ad libitum* fed mice during the light phase, let alone induce oxytocin release within white adipose tissue directly, a lack of effect on RER is perhaps unsurprising.

Data presented here supports a role of Oxt^PVH^ neurons in regulating energy expenditure. An increase in energy expenditure and body temperature, without a significant rise in locomotor activity, is most likely explained by an increase in brown adipose-mediated thermogenesis. Sutton et al. (24) reported a small but significant increase in locomotor activity following Oxt^PVH^ neuron activation and no effect on temperature (or expression of uncoupling protein 1) in interscapular brown adipose tissue. The literature supports a role for oxytocin neurons in the thermogenic response to cold exposure (23), likely driven by Oxt^PVH^ projections to the rostral medullary raphe (26). Oxytocin is undoubtedly capable of modulating autonomic output (1). As evidenced by loss of function studies (22, 23), oxytocin neurons play an obligate role in maintaining different aspects of energy expenditure, particularly in response to environmental challenge (e.g. obesogenic diet, cold exposure).

Having identified oxytocin fibers and oxytocin receptors in the BNST, we decided to investigate whether a PVH to BNST projection could modulate consumption. The BNST is considered part of the “extended amygdala” and, thus, has been implicated in the neuroendocrine control regulation of complex behaviors including stress, reward and appetite (54, 90, 91). The BNST exerts powerful control over motivational feeding via GABAergic projections to the LH and VTA (92, 93), whereby activation of BNST GABAergic projections stimulate food intake. Given the subtle effects of Oxt^PVH^ chemogenetic modulation in our previous studies, we reasoned that selectively activating a single projection of Oxt^PVH^ neurons may have a limited effect on chow intake. Therefore, we decided to investigate the ability of Oxt^PVH^ neurons to specifically reduce sucrose intake. Oxytocin preferentially suppresses the intake of sweet-tasting, high-carbohydrate diets and oxytocin knock-out mice over consume palatable sucrose, but not palatable lipid solutions (8, 44, 45, 47, 51–53, 94). Accordingly, we found that optogenetic stimulation of Oxt^PVH^ fibers selectively within the BNST reduced sucrose intake in otherwise sated mice, recapitulating the ability of oxytocin to reduce intake of sweet carbohydrates. Since Oxtr-positive neurones in the BNST appear themselves to be GABAergic (74, 95), oxytocin may cause local inhibition of projection neurons to affect behavior (73, 96). Whether oxytocin inhibits LH- and VTA-projecting neurons in the BNST requires further investigation; however we provide a clear indication that the BNST may be involved in oxytocin’s ability to preferentially reduce sucrose intake.

## Conclusions

The evidence for a role of oxytocin neurons in modulating different aspects of energy balance has built up over the last 40 years, but recently has been challenged following utilization of the latest genetic tools. The ability to selectively manipulate oxytocin neurons in freely behaving animals is game changing. However, there are important technical considerations to accommodate since native oxytocin likely exerts modulatory effects over several brain circuits and may function in a permissive manner. We have demonstrated that Oxt^PVH^ neurons can increase energy expenditure, reduce normal night-time feeding and regulate sucrose intake, specifically. The action of Oxt^PVH^ neurons differs somewhat from experimentally applied oxytocin, as they appear to exert subtle modulatory effects that are context specific. Such studies of selective neuronal populations have relevance for understanding not only the physiology of native oxytocin signaling, but also the etiology of oxytocin deficiency syndromes. There is still much to learn about the physiological role of oxytocin-containing cells. The experiments performed here highlight subtleties and nuanced approaches required in future studies investigating this intriguing population.

## Data Availability

The raw data supporting the conclusions of this article will be made available by the authors, without undue reservation.
