# Supplementary material for: Paraventricular oxytocin neurons impact energy intake and expenditure: projections to the bed nucleus of the stria terminalis reduce sucrose consumption
Source: Front Endocrinol (Lausanne). 2024 Sep 2;15:1449326. doi: 10.3389/fendo.2024.1449326 (PMC11402739; doi:10.3389/fendo.2024.1449326)
Supplement: Supplementary file 1 [file DataSheet1.pdf]

Paraventricular oxytocin neurons impact energy intake and expenditure: projections to the bed nucleus of the stria terminalis reduce sucrose consumption

Amy A. Worth, Claire H. Feetham, Nicole A. Morrissey, Simon M. Luckman*.

Supplementary Table 1

Treatment	# PVH Oxt^{+}	# PVH Oxt^{+} cFos^{+}	% Oxt^{PVH} activation	# animals
Fasted	56.1 ± 12.5	0.8 ± 0.4	1.7 ± 0.8	4
Fed	52.7 ± 7.6	10.4 ± 2.0	20.5 ± 4.7	4
Oral Saline	18.2 ± 3.5	0.9 ± 0.5	7.3 ± 4.5	6
Oral Lipid	17.3 ± 3.1	3.4 ± 1.1	17.6 ± 3.7	6
IP Vehicle	58.6 ± 5.8	3.2 ± 0.8	5.6 ± 1.4	5
IP CCK	60.0 ± 2.9	9.4 ± 0.8	15.7 ± 1.2	5
IP NaCl	49.6 ± 12.2	3.3 ± 0.9	14.3 + 8.8	6
IP LiCl	59.9 ± 8.5	25.4 ± 4.4	41.3 ± 6.5	6

Table S1. Average number of Oxt^{PVH} cells and cFos-expressing Oxt^{PVH} cells counted per section and percentage activation of Oxt^{PVH} neurons following each treatment in wild-type male mice. All values are mean ± SEM.

Supplementary Table 2

Treatment	#PVH Oxt^{+}	# PVH mCherry^{+}	# PVH cFos^{+}	# PVH Oxt^{+} mCherry^{+}	# PVH mCherry^{+} cFos^{+}	# animals
IP Vehicle	81.3 ± 21.1	53.3 ± 11.3	12.5 ± 4.2	48.0 ± 9.5	5.0 ± 2.0	4
IP CNO	90.3 ± 17.3	66.8 ± 9.9	64.5 ± 6.4	59.3 ± 12.5	50.0 ± 12.0	4

Table S2. Average number of Oxt, mCherry, cFos, mCherry-expressing Oxt and cFos-expressing mCherry cells counted per section in the PVH of *Oxt*$^{Cre:hM3Dq}$ following either IP Vehicle or IP CNO. All values are mean ± SEM.

Supplementary Figure 1

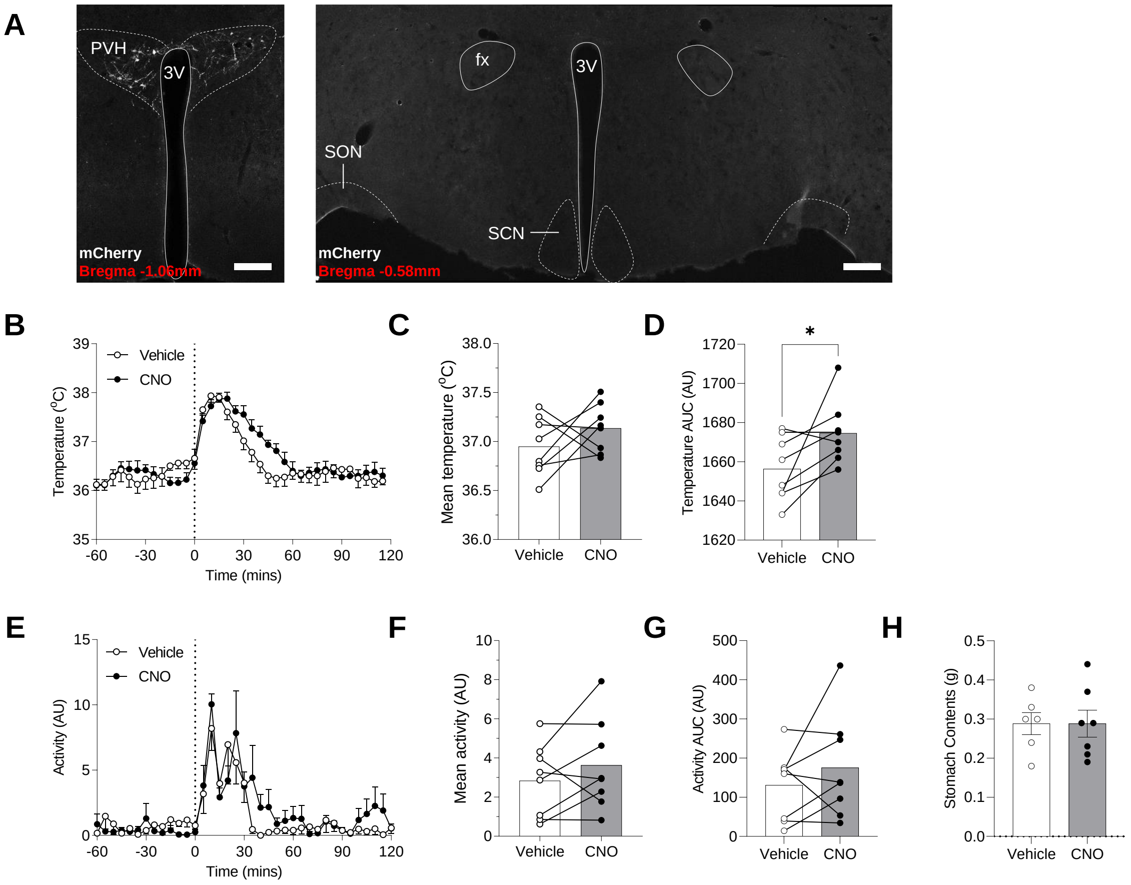

Figure S1. **(A)** Representative image of mCherry staining in a transfected PVH of an Oxt^{Cre} mouse injected with AAV-DIO-hM3Dq-mCherry and lack of staining in the supraoptic nucleus of the same animal. 3V: third ventricle. fx: fornix. PVH: paraventricular nucleus of the hypothalamus. SCN: suprachiasmatic nucleus. SON: supraoptic nucleus. Scale bar 250 µm. **(B)** Temperature following IP injection of vehicle or CNO in male $Oxt^{Cre:hM3Dq}$ mice. Data presented as mean ± SEM, mixed-effects analysis of variance with Sidak's multiple comparisons *post hoc* test (n = 8). **(C)** Average temperature between 0 hr and 1 hr, compared between different treatments (vehicle vs. CNO). $P > 0.05$; paired *t*-test (n = 8). **(D)** Area under the curve (AUC) for temperature between 15 min and 60 min. * $P < 0.05$; paired *t*-test (n = 8). **(E)** Activity following IP injection of vehicle or CNO in $Oxt^{Cre:hM3Dq}$ mice. Data presented as mean ± SEM, mixed-effects analysis of variance with Sidak's multiple comparisons *post hoc* test (n = 8). **(F)** Average activity between 10 min and 60 min, compared between different treatments (vehicle vs. CNO). $P > 0.05$; paired *t*-test (n = 8). **(G)** Area under the curve (AUC) for activity between 10 min and 60 min. $P > 0.05$; paired *t*-test (n = 8). **(H)** Gastric emptying was assessed by measuring stomach contents following oral gavage of a semi-liquid meal in

Oxt$^{Cre:hM3Dq}$ mice previously injected IP with either vehicle or CNO. P > 0.05; unpaired *t*-test (n = 6/7).

Supplementary Figure 2

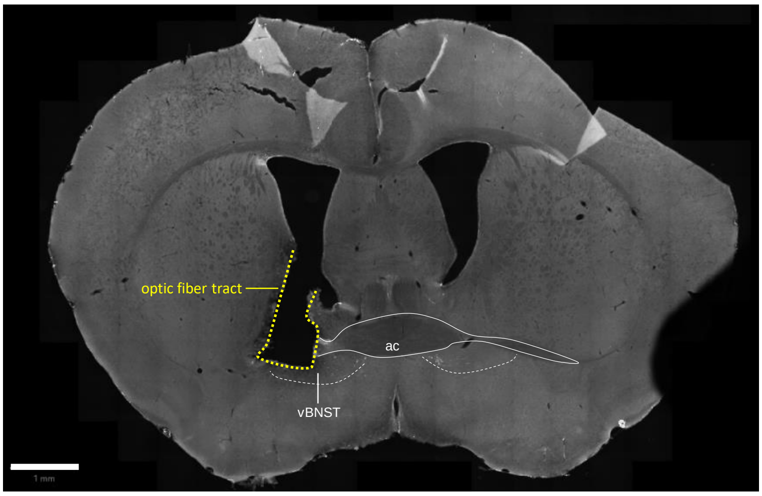

Figure S2. **(A)** Representative image of optic fiber placement (yellow dotted line) in an *Oxt*$^{Cre:ChR2}$ animal. ac: anterior commissure. vBNST: ventral bed nucleus of the stria terminalis. Scale bar 1 mm.
